# Evaluating Middle Ear Absorbance and Resonant Frequency in Patulous Eustachian Tube Patients Using Wideband Tympanometry

**DOI:** 10.1097/ONO.0000000000000076

**Published:** 2025-08-27

**Authors:** Iori Kusaka, Ryoukichi Ikeda, Masato Suzuki, Daiki Onodera, Aya Katsura, Jun Suzuki, Kiyoto Shiga

**Affiliations:** 1 Department of Otolaryngology and Head and Neck Surgery, Iwate Medical University School of Medicine, Iwate, Japan; 2 Department of Otolaryngology and Head and Neck Surgery, Tohoku University Graduate School of Medicine, Sendai, Japan.

**Keywords:** Middle ear absorbance, Resonant frequency, Sonotubometry, Tympanic membrane movement

## Abstract

**Objective:**

This study aimed to investigate the utility of wideband tympanometry (WBT) in diagnosing patulous Eustachian tube (PET) by examining differences in middle ear absorbance and resonant frequency between PET patients and controls.

**Study Design:**

This is a retrospective observational study.

**Setting:**

This study was conducted at Iwate Medical University Hospital.

**Subjects and Methods:**

A review of medical records identified 25 ears of 19 PET patients and 18 ears of 9 patients with sensorineural hearing loss or vertigo as a control group. Diagnosis of PET was based on Japan Otological Society criteria. WBT was conducted across frequencies of 226–8000 Hz, measuring ambient pressure absorbance ratios and peak pressure absorbance ratios, as well as resonant frequency (RF) values.

**Results:**

The PET group demonstrated significantly lower absorbance at 1000 Hz (*P* = 0.006) compared with the control group, with no significant differences observed at other frequencies. The RF was significantly reduced in the PET group compared with controls (*P* < 0.001), indicating alterations in middle ear mechanics associated with PET. The Valsalva maneuver had no significant effect on absorbance values across frequencies.

**Conclusion:**

WBT is a valuable diagnostic tool for PET, with significant reductions in 1000 Hz absorbance and resonant frequency in PET patients. These parameters may serve as markers for PET, aiding in its differentiation from other middle ear conditions.

The Eustachian tube (ET) typically remains closed at rest, opening transiently during swallowing to perform essential functions, including ventilating the middle ear, facilitating secretion drainage, and shielding the middle ear from direct sound transmission and nasopharyngeal secretions. Patulous Eustachian tube (PET) is a dysfunction characterized by impaired ET closure, resulting in symptoms such as autophony of one’s voice and breathing sounds, along with aural fullness (1,2). PET is frequently linked to weight loss (3) and may also occur in association with conditions such as pregnancy, neuromuscular disorders, cerebrovascular diseases, temporomandibular joint dysfunction, and craniofacial anomalies. Despite decades of efforts to establish objective diagnostic methods for PET, no single test has yet proven reliably effective for diagnosis (4,5).

Wideband tympanometry (WBT) utilizes click stimuli, as opposed to traditional tympanometry, which typically relies on a single-probe tone (6). This feature enables WBT to measure middle ear immittance over a broad frequency spectrum, from 226 to 8000 Hz, under both ambient conditions and varied external auditory canal pressures. In WBT, the acoustic absorbance parameter quantifies the sound energy absorbed by the middle ear, whereas the reflectance value denotes the amount of sound energy reflected back from the tympanic membrane. An additional critical parameter is the resonance frequency (RF), which identifies the frequency at which the mass and stiffness elements of the middle ear achieve equilibrium. At the RF, friction predominantly influences the middle ear, facilitating maximal sound energy transmission compared with other frequencies (7,8).

Changes in middle ear absorbance may occur in patients with PET; however, to the best of our knowledge, no studies have investigated the findings of WBT in PET patients. This study aimed to investigate WBT parameters in PET patients and evaluate the usefulness to diagnose PET.

## METHODS

### Patients

We retrospectively retrieved the medical records of PET and non-PET patients who visited Iwate Medical University Hospital between January 2023 and July 2024. Twenty-five ears of 19 PET patients (15 male and 4 female subjects, aged 15–86, median 50.6 years) and 18 ears of 9 patients (5 male and 4 female subjects, aged 39–83, median 62.4 years) with SNHL or vertigo without ET dysfunction findings were included in this study, with the latter group as the control. This study was approved by the Iwate Medical University Hospital Institutional Review Board.

### Diagnosis of PET

The diagnosis of PET was based on the criteria established by the Japan Otological Society (9), which are outlined below: (1) The presence of aural symptoms such as autophony and/or aural fullness. (2) Symptom relief when the ET is closed (either by assuming a supine or prone position or by artificially blocking the pharyngeal orifice of ET with methods such as using cotton or jelly). (3) Objective evidence of an open ET (such as tympanic membrane movement during respiration, findings from Tubo-Tympano-Aerodynamic-Graphy [TTAG], or sonotubometry) (10). A definite PET diagnosis is made if all 3 criteria (1 + 2 + 3) are fulfilled, while a possible PET diagnosis is considered if only 2 criteria (1 + 2 or 1 + 3) are met. In this study, all PET patients were diagnosed with definite PET.

### Wideband Tympanometry

WBT was conducted across octave frequencies ranging from 226 to 8000 Hz (covering 107 distinct frequencies) at an intensity level of 100 dB sound pressure level using the Interacoustics Titan (version 3.1; IMP440, Denmark) in a quiet environment. The test commenced after inserting the appropriate probe into the participants’ external auditory canal. The collected data were stored in the system’s database as an .xls file. WBT parameters—such as RF, ambient pressure absorbance ratios (APAR), and peak pressure absorbance ratios (PPAR) at 226, 500, 1000, 2000, 4000, and 8000 Hz—were analyzed.

### Statistical Analysis

Absorbance data were compared using the Mann-Whitney U test for nonparametric data. The primary outcome measures were the differences in absorbance at specific frequencies and changes in RF under different conditions. Receiver operating characteristic (ROC) curve was generated. All statistical analyses were conducted using SPSS version 27 (IBM, Chicago, IL, USA). A *P*-value of <0.05 was considered statistically significant.

## RESULTS

### Absorbance

**FIG. 1.**
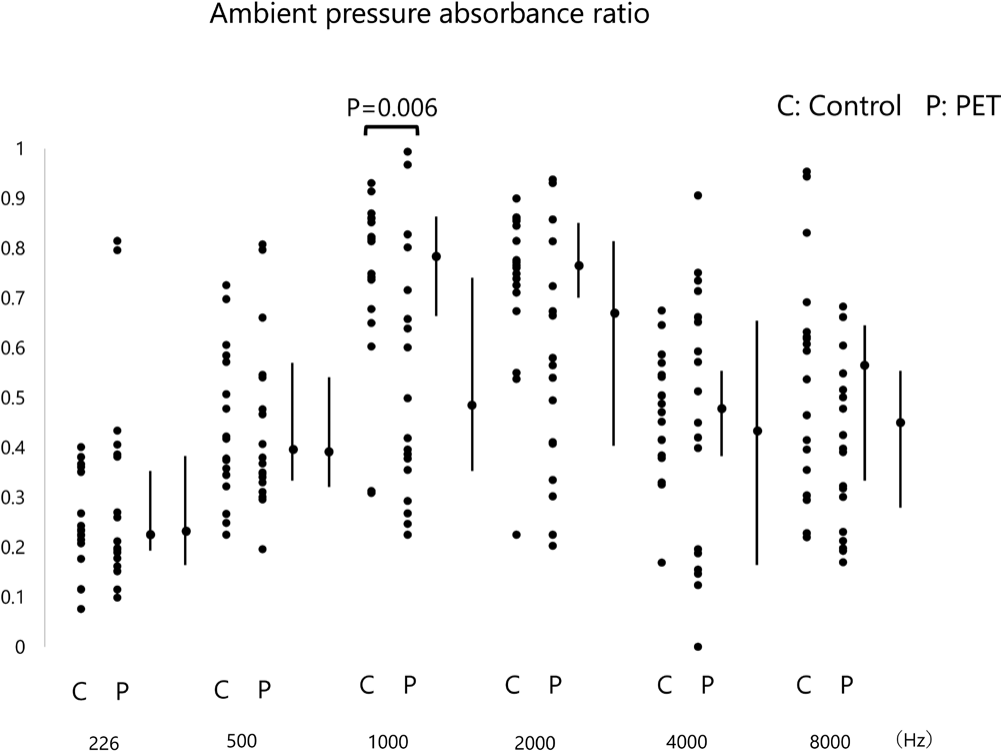
Ambient pressure absorbance ratios across frequencies in control and PET groups. Ambient pressure absorbance ratios are displayed at frequencies of 226, 500, 1000, 2000, 4000, and 8000 Hz for both the control (C) and PET (P) groups. Ambient pressure absorbance ratios are presented as median (interquartile range).

**FIG. 2.**
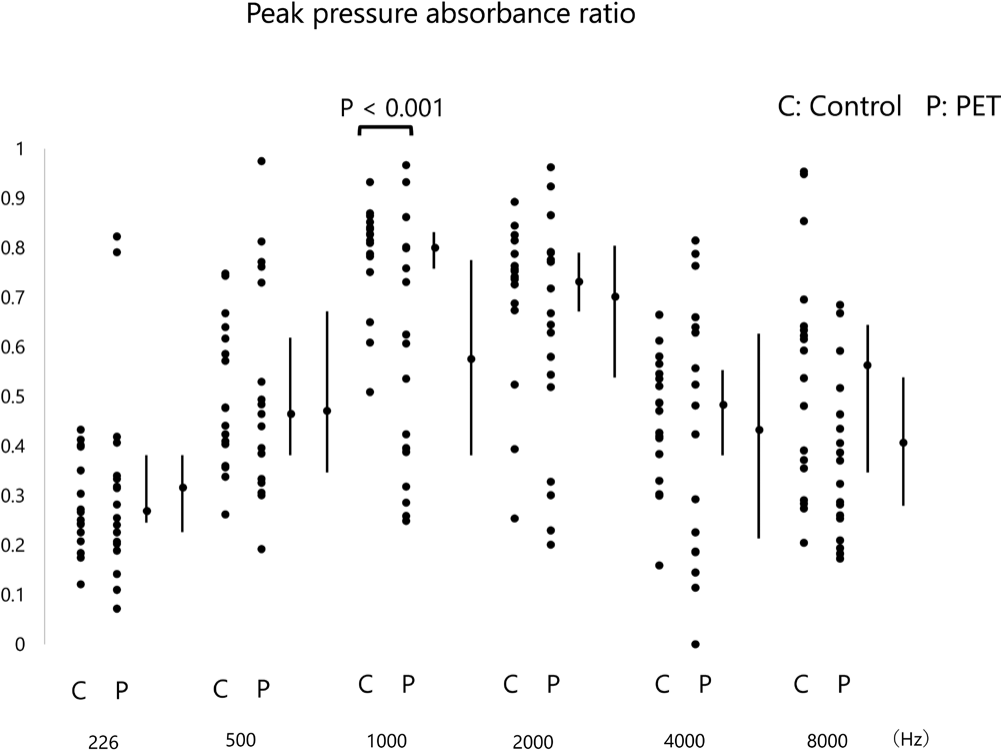
Peak pressure absorbance ratios across frequencies in control and PET groups. Peak pressure absorbance ratios at 226, 500, 1000, 2000, 4000, and 8000 Hz for control (C) and PET (P) groups. Peak pressure absorbance ratios are presented as median (interquartile range).

In comparing the absorbance values, we observed no differences between 2 groups in terms of 226, 500, 2000, 4000, and 8000 Hz APAR and PPAR values (*P* > 0.05). In the PET group, APAR (0.486, range: 0.352–0.731) (Fig. 1) and PPAR (0.579, range: 0.375–0.798) (Fig. 2) were significantly lower at 1000 Hz compared with the control group (0.782, range: 0.671–0.860 and 0.821, range: 0.775–0.855) (*P* = 0.006 and *P* < 0.001, respectively). The ROC curve analysis revealed an area under the curve (AUC) of 0.513 (95% confidence interval [CI]: 0.329–0.696) for APAR (Fig. 6A) and an identical AUC of 0.513 (95% CI: 0.329–0.696) for PPAR (Fig. 6B).

**FIG. 3.**
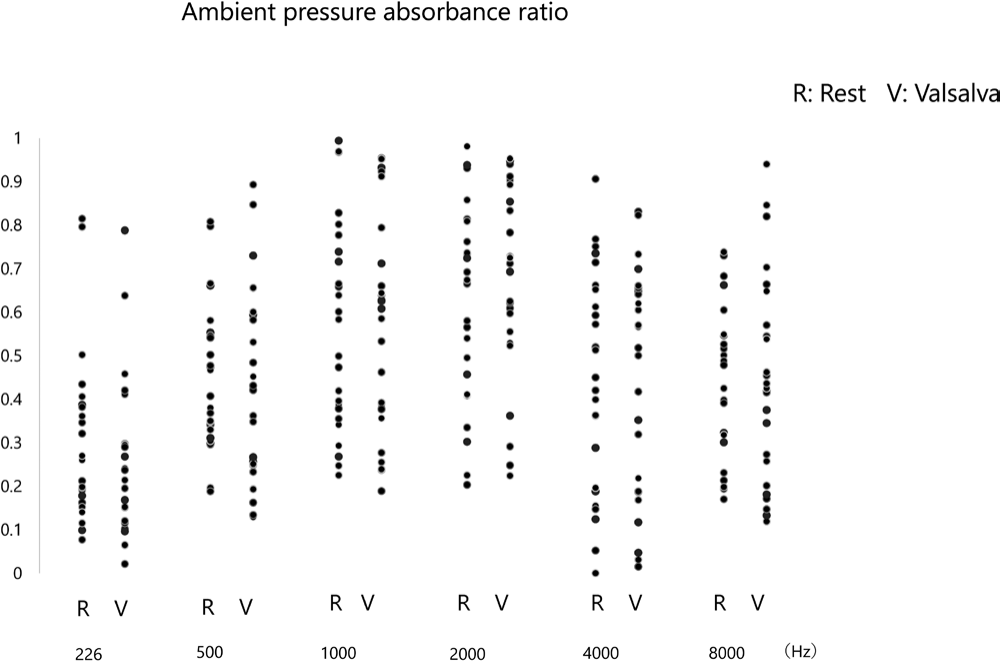
Ambient pressure absorbance ratios in rest and valsalva conditions of the PET patients. Ambient pressure absorbance ratios measured at rest (R) and after performing the Valsalva maneuver (V) at 226, 500, 1000, 2000, 4000, and 8000 Hz.

**FIG. 4.**
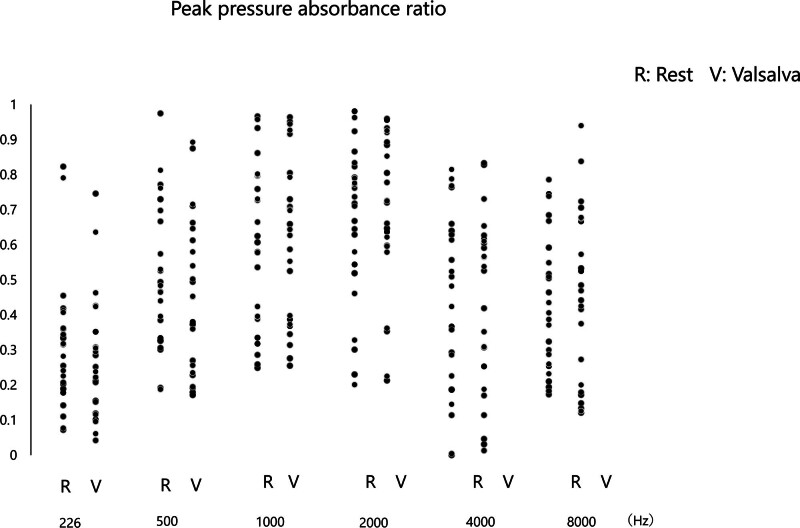
Peak pressure absorbance ratios in rest and valsalva conditions of the PET patients. Peak pressure absorbance ratios in rest (R) and valsalva (V) conditions at frequencies of 226, 500, 1000, 2000, 4000, and 8000 Hz.

**FIG. 5.**
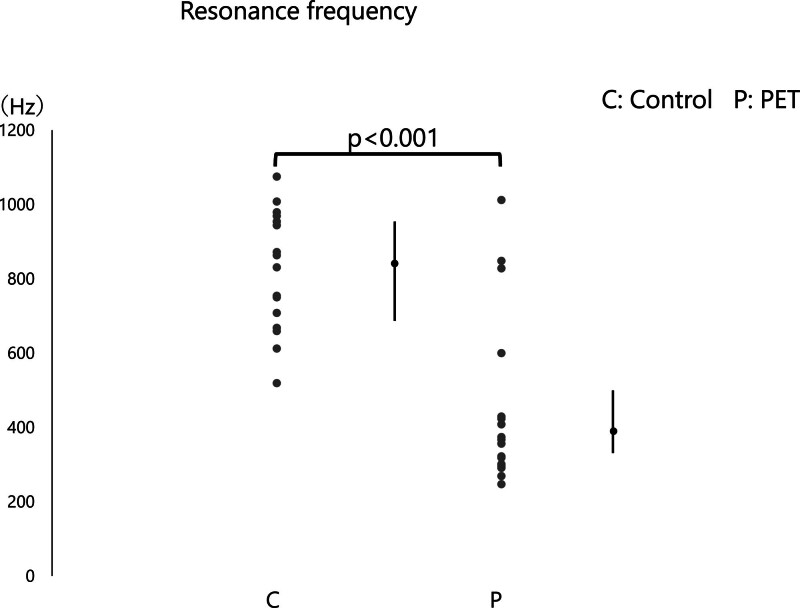
Resonant frequency comparison between control and PET groups. Median resonant frequency (Hz) values are compared between the control group (C) and the patulous Eustachian tube (PET) group (P). Resonant frequency is presented as median (interquartile range).

**FIG. 6.**
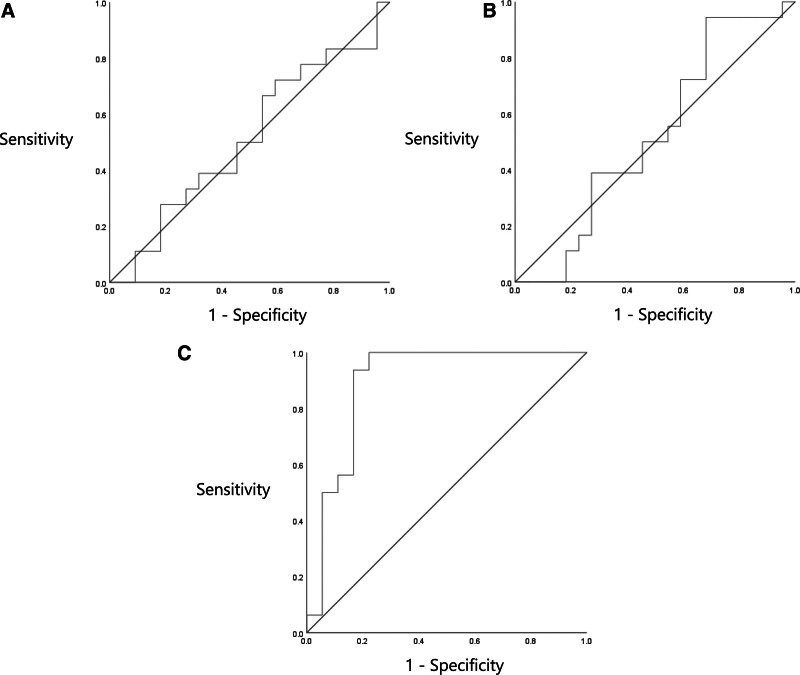
Receiver operating characteristics (ROC) curve for Ambient pressure absorbance ratios, peak pressure absorbance ratios, and resonant frequency. *A*, Ambient pressure absorbance ratios. *B*, Peak pressure absorbance ratios. *C*, Resonant frequency.

### Comparison of Rest and Valsalva Conditions

The Valsalva maneuver did not produce significant changes in APAR (Fig. 3) and PPAR (Fig. 4) for the all group across all frequencies compared with the values at their rest condition.

### Resonant Frequency Analysis

The mean RF was 847.0 Hz (range: 678–965.3 Hz) in the normal and 361.5 Hz (range: 299.8–471.8 Hz) in the PET group. The RF analysis revealed that PET group exhibited a significant reduction compared with the control group (*P* < 0.001) (Fig. 5). ROC curve analysis identified 559.5 Hz as the optimal diagnostic cutoff value for RF, with a sensitivity of 0.938 and specificity of 0.833 (AUC = 0.523, 95% CI: 0.342–0.705) (Fig. 6C).

## DISCUSSION

This study aimed to evaluate the utility of WBT in diagnosing PET and explore its ability to identify differences in middle ear absorbance and RF between PET patients and control subjects. Our findings revealed significant differences in middle ear absorbance at specific frequencies, particularly at 1000 Hz, between PET and control groups. Additionally, RF in PET patients was significantly lower than in controls, underscoring WBT’s ability to detect mechanical alterations in the middle ear associated with PET.

### Absorbance Differences

The significant reduction in absorbance at 1000 Hz in PET patients compared with controls is a key finding. This finding suggests that PET may lead to specific changes in middle ear mechanics, particularly at mid-range frequencies. However, the analysis using the ROC curve did not yield a significant AUC, indicating limited effectiveness. The tympanic membrane retraction commonly seen in PET patients, particularly those with the sniffing type (11), may explain this finding. Sniffing induces negative pressure in the middle ear (12,13), which may reduce the mobility of the tympanic membrane, resulting in lower absorbance at these frequencies. Notably, other frequencies, such as 226, 500, 2000, 4000, and 8000 Hz, did not show significant differences, indicating that absorbance changes in PET patients may be frequency-specific and related to the dynamic properties of the middle ear at certain frequencies. Previous studies have suggested that traditional tympanometry, which uses a single low-frequency probe tone, is less sensitive to detecting subtle middle ear changes, particularly in conditions such as PET (14). The multifrequency approach of WBT allows for a more comprehensive assessment of middle ear function across a wider range of frequencies, which could explain why our study was able to detect significant differences at 1000 Hz. However, in this study, the ROC curve analysis did not yield a significant AUC, nor was it possible to establish an effective cutoff value. Therefore, alternative loading methods, such as sniffing or other forms of negative pressure application, as well as the use of the Toynbee maneuver, may be useful. Additionally, investigating correlations with the degree of ET patency using other modalities—such as upright-position CT imaging—may yield further valuable insights.

## RESONANT FREQUENCY

The significant reduction in RF in PET patients compared with controls suggests that PET impacts the mechanical properties of the middle ear. Acoustic resonance occurs when a vibrating object matches the phase of incoming sound waves, resulting in amplified vibrations compared with nonresonant states. The specific frequency at which an object resonates is determined by its unique mass and stiffness. At this RF, mass-reactance and stiffness-reactance counterbalance perfectly, minimizing impedance and eliminating reactance’s effect on the system. Mathematically, this state is represented as Xm − Xs = 0 and a phase of zero degrees (15). In systems where stiffness dominates, resonance occurs at higher frequencies, whereas in mass-dominated systems, resonance shifts to lower frequencies. RF depends on the ratio of stiffness to mass. Adjusting these parameters alters RF: increasing stiffness raises RF, while increasing mass lowers it. This principle is evident in tuning guitar strings, where altering string tension changes stiffness, tuning strings to higher frequencies when tension is increased and to lower frequencies when tension is relaxed. Similarly, the density (mass) of strings influences frequency: thicker strings resonate at lower frequencies, while thinner, lighter strings vibrate at higher frequencies (16). This finding is consistent with the clinical understanding that PET leads to excessive openness of the ET, resulting in a less stiff middle ear system that transmits sound differently than in normal ears. This reduction in RF is in line with previous studies that have demonstrated changes in middle ear stiffness in other pathologies, such as superior semicircular canal dehiscence (SSCD) (17), enlarged vestibular aqueducts (18), and Meniere’s disease (19,20), which also show decreased RF. The SSCD presents with overlapping symptoms with PET, including aural fullness and voice autophony, making differential diagnosis essential (1,21). It is conceivable that decreased resonance—reflecting changes in stiffness and mass reactance—may be associated with these aural symptoms; however, further investigation is warranted to clarify this relationship. The fact that PET shows similar trends highlights the broader utility of WBT in diagnosing and differentiating middle ear conditions based on mechanical impedance changes.

## CLINICAL IMPLICATIONS

The findings of this study have important clinical implications for the diagnosis and management of PET. WBT offers a noninvasive, efficient, and reliable method for assessing middle ear function, making it a valuable diagnostic tool in settings where PET is suspected. The frequency-specific changes in absorbance, particularly at 1000 Hz, provide a potential diagnostic marker for PET, which could be used alongside other diagnostic tools, such as tubomanometry, TTAG, and sonotubometry (5), to improve diagnostic accuracy. Furthermore, the ability to detect RF changes in PET patients suggests that WBT could be used to monitor disease progression or response to treatment.

## LIMITATION

Despite the promising findings, this study has several limitations. The sample size, particularly in the sniffing PET group, was relatively small, which may limit the generalizability of the results. In particular, it would have been beneficial to perform logistic regression analysis; however, this was not feasible due to the limited number of cases in each group. Future studies should aim to include larger and more diverse patient populations to validate these findings. Additionally, while WBT provides valuable data on middle ear mechanics, it does not directly measure ET function. Combining WBT with other diagnostic tools, such as tympanic membrane movement, tubomanometry, TTAG, sonotubometry, and imaging tool (22), could provide a more comprehensive assessment of PET. Further research is also needed to explore the longitudinal impact of PET on middle ear function and to determine whether WBT can predict the progression of the condition or the response to treatment. Studies investigating the correlation between WBT findings and symptom severity would be particularly useful in refining the diagnostic criteria for PET.

## CONCLUSION

WBT has shown significant potential in detecting middle ear abnormalities in PET, particularly in differentiating between subtypes of the condition. The WBT, particularly the assessment of RF, holds potential as a supportive tool in the diagnosis of PET. Further research will be essential in expanding our understanding of PET and in optimizing the use of WBT in clinical practice.

## FUNDING SOURCES

This study was supported by Grant-in-Aid for Scientific Research (C) 23K08993.

## CONFLICT OF INTEREST STATEMENT

None declared.

## DATA AVAILABILITY STATEMENT

Data are not publicly available due to ethical reasons. Further enquiries can be directed to the corresponding author.
